# Study protocol of a cluster randomised controlled trial investigating the effectiveness of a tailored energy balance programme for recent retirees

**DOI:** 10.1186/1471-2458-6-293

**Published:** 2006-12-06

**Authors:** Andrea Werkman, Albertine J Schuit, Lydia Kwak, Stef PJ Kremers, Tommy LS Visscher, Frans J Kok, Evert G Schouten

**Affiliations:** 1 Division of Human Nutrition, Wageningen University, PO Box 8129, 6700 EV, Wageningen, the Netherlands; 2 National Institute for Public Health and the Environment, PO Box 1, 3720 BA, Bilthoven, the Netherlands; 3 Department of Human Biology, Maastricht University, PO Box 616, 6200 MD, Maastricht, the Netherlands; 4 Department of Health Education and Promotion, Maastricht University, PO Box 616, 6200 MD, Maastricht, the Netherlands; 5 Institute for Health Sciences, Vrije Universiteit, De Boelelaan 1085, 1081 HV, Amsterdam, the Netherlands

## Abstract

**Background:**

People in transitional life stages, such as occupational retirement, are likely to gain weight and accumulate abdominal fat mass caused by changes in physical activity and diet. Hence, retirees are an important target group for weight gain prevention programmes, as described in the present paper.

**Methods/Design:**

A systematic and stepwise approach (Intervention Mapping) is used to develop a low-intensity energy balance intervention programme for recent retirees. This one-year, low-intensity multifaceted programme aims to prevent accumulation of abdominal fat mass and general weight gain by increasing awareness of energy balance and influencing related behaviours of participants' preference. These behaviours are physical activity, fibre intake, portion size and fat consumption. The effectiveness of the intervention programme is tested in a cluster randomised controlled trial. Measurements of anthropometry, physical activity, energy intake, and related psychosocial determinants are performed at baseline and repeated at 6 months for intermediate effect, at 12 months to evaluate short-term intervention effects and at 24 months to test the sustainability of the effects.

**Discussion:**

This intervention programme is unique in its focus on retirees and energy balance. It aims at increasing awareness and takes into account personal preferences of the users by offering several options for behaviour change. Moreover, the intervention programme is evaluated at short-term and long-term and includes consecutive outcome measures (determinants, behaviour and body composition).

## Background

This study is performed as part of the Netherlands Heart Foundation 'Netherlands Research programme for weight Gain prevention' (NHF-NRG). This multidisciplinary programme aims to gain insight into behavioural determinants of weight gain and to identify potentially effective methods and strategies for the prevention of weight gain in distinct target groups: adolescents, young adults and recent retirees [1].

Overweight and obesity are associated with chronic conditions such as diabetes, hypertension, cardiovascular diseases and certain types of cancer, and thus considered a major public health concern [2]. Many attempts have been made to treat overweight and obesity and although these attempts show short term weight loss in most subjects, weight is often regained after cessation of the intervention [3,4]. Therefore, it has been suggested that prevention of weight gain in the general population may be a more effective strategy for addressing the overweight and obesity epidemic [5-11]. However, studies testing weight gain prevention programmes among adults are limited and often not successful. A review by Hardeman *et al *[12] regarding interventions to prevent weight gain shows that the interventions exhibited various degrees of effectiveness. Furthermore, it is not clear what elements of the interventions are associated with increased effect size, since only one of the five studies that involved an RCT design reports a significant effect on weight. The authors plead for more objective measures of physical activity and diet in future studies and for longer periods of follow-up [12].

Programmes to prevent weight gain should focus on the balance between physical activity and energy intake from foods, also referred to as energy balance [13]. Target groups are segments of the population at elevated risk of weight gain. These are often populations going through transitional life stages [1,10,14], leading to changes in daily routine. Occupational retirement is such a transitional life stage. Retirees lack work-related physical activity, which may not be compensated after retirement. Moreover, they have increased access to food and more opportunity for eating. Since, apart from retirement, ageing itself can also lead to increased fat mass and to decreased skeletal muscle mass [15,16], retirees are an important group for weight gain prevention.

This paper presents the development of the intervention programme and the study design of the cluster randomised controlled trial, called the Wageningen Approach against fat Accumulation and weight Gain (WAAG-Study). The main aim of the programme is to prevent weight gain, in particular accumulation of abdominal fat mass in recent retirees by increasing awareness of energy balance and subsequently adapting energy balance-related behaviours according to participants' preferences.

## Methods/Design

### Participants and recruitment

Participants for this study are recruited from pre-retirement workshops offered by employers to approximately 10% of Dutch retirees. During such a five-day workshop several topics are discussed in order to prepare retirees for the new phase in life, e.g. changes in the household after retirement, health and vitality, and their new role in society. Inclusion criteria for the present study are: age between 55–65 years, recently retired, defined as maximal six months before or after date of retirement at inclusion, apparently healthy and not undergoing any medical treatment that might affect the outcome measures. Written informed consent from participants is obtained upon enrolment. The study protocol is approved by the Medical Ethics Committee of Wageningen University.

### Study design

We randomise all participants from one workshop together rather than individually because we fear adoption of the intervention programme by individuals in the control group. Thus, clusters of workshop participants are allocated to either the intervention or control group. Allocation is performed the week following baseline examination by an independent person and taking into account the number participants per workshop and the number of included clusters per week.

We hypothesise that waist circumference in the control group will increase with 0.5 cm per year (standard deviation = 1.3 cm) while it will remain stable among subjects in the intervention group. This is based on data obtained from a middle-aged (56–65 years) Dutch population from the 'Doetinchem Cohort' (National Institute for Public Health and the Environment, Bilthoven, the Netherlands). Calculations reveal a sample size of a total of 400 individuals, taking into account 80% power, cluster randomisation [17] with an estimated design effect of 20% [18] and assuming a drop out of 25%.

### Theoretical basis of the intervention programme

The intervention programme is developed according to the Intervention Mapping protocol that facilitates a systematic, stepwise process of designing health behaviour interventions [19]. Basically, it comprises of a needs assessment of the study population, an inventory of factors that influence health behaviour, a definition of the main aim, a subdivision into practicable behaviours, a linkage to determinants of behaviour, a translation into methods and practical strategies and the development of a detailed program plan.

**Figure 1:**
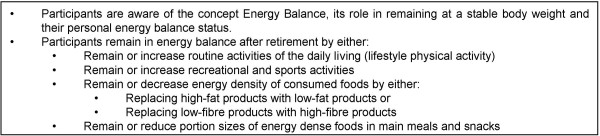
Programme objectives of the intervention programme.

The main aim of the intervention programme is to prevent weight gain and in particular accumulation of abdominal fat mass. Practicable intermediate steps to reach this aim are increased awareness of energy balance and related behaviours and subsequently prevention of unfavourable change in diet and/or physical activity. For the purpose of intelligibility, energy balance is defined as the equilibrium between energy intake from the diet and energy expenditure from physical activity. Specific energy balance-related behaviours that are identified for the present study are depicted in figure 1 and elucidated below.

The intervention programme focuses on two domains of physical activity: daily routine physical activity and recreational/sport activities. Daily routine physical activity is of importance, since retirees lack work-related physical activity that may not be compensated after retirement. Therefore, retirees need to be aware of opportunities for daily physical activity, such as household activities and active transportation [20,21]. Furthermore, retirees have more time available and thus recreational and sport activities, e.g. bicycling and walking, are also incorporated in the programme.

Two strategies related to energy-density are included in the programme: replacement of high-fat foods with low-fat foods [22-27] and replacement of low-fibre foods with high-fibre foods [26,28-31]. The intervention programme also focuses on the reduction of portion sizes of energy dense foods during the main meals of the day and during snacking [32,33].

The behaviours mentioned above are further linked to their matching psychosocial determinant: knowledge, awareness, attitude, perceived self-efficacy, and habit. In our study, knowledge is further subdivided in nutritional knowledge [34], knowledge of fibre-rich products [35] and knowing health benefits of a healthy diet and physical activity. Previous studies have shown that increasing awareness of personal physical activity and dietary behaviours is important [36,37] as well as attitude [38-41]. Furthermore, perceived self-efficacy has been shown to be a predictor for the consumption of fruits, vegetables and low fat diets [42] and may affect the consumption of large portion sizes [43]. Finally, habit is a factor that needs to be taken into account, since dietary behaviour has often become a habit since childhood [38,44,45] and it may also influence behavioural choices regarding physical activity [46,47].

### Intervention materials

**Figure 2:**
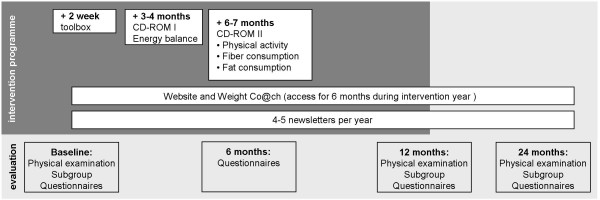
Overview of the intervention programme (dark grey box) and timeline of the evaluation plan (bottom part).

Based on the linkage of determinants of energy balance-related behaviour to methods and strategies, existing materials are identified and new materials are developed. This resulted in our energy balance programme, that has a low-intensity, multifaceted character and is disseminated to the intervention group over a period of one-year (see table 1 for a detailed description of the programme). Figure 2 shows the moments of distribution of the materials. The first element in the programme is a toolbox that contains an instruction leaflet and several instruments to increase awareness and knowledge of personal status of physical activity, dietary intake and weight. The second element is a CD-ROM that contains stage-matched computer tailored feedback on current energy balance status. This feedback also incorporates attitude and self-efficacy [48,49]. The outcome of the test and/or personal preference can be employed in the subsequent element, a second CD-ROM. This also contains a computer tailored programme and offers four different options for feedback on personal behaviour:

1. Feedback on total physical activity, including daily routine and sport activities;

2. Feedback on fibre intake;

3. Feedback on portion sizes of energy dense foods (hot or cold meals, snacks or beverages);

4. Feedback on fat intake (hot or cold meals, snacks).

Thus, participants can choose to receive individual feedback on one or more options. The feedback consists in all cases of a letter that states the current status compared to the norm (e.g. the norm for fat intake in the Netherlands, or minutes of physical activity per day). Participants can print this letter and can also choose to formulate an action plan for physical activity and can use the low-fat recipes that are available on the second CD-ROM.

Apart from the tool box and CD-ROMs, participants of the intervention group receive printed newsletters with information on the study and encouragements and prompts to use the materials and choose another option from the second CD-ROM. They also have access to the study's website, with login facilities for additional information and the Weight Co@ch, an interactive programme developed by TNO Quality of Life [50].

Subjects of the control group receive newsletters with general information only, e.g. announcements for art exhibitions and have limited access to the website.

### Outcome measurements

**Figure 3:**
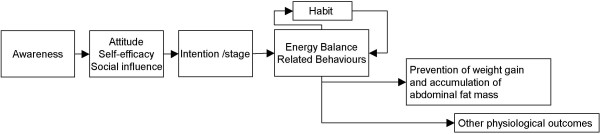
Evaluation plan (adapted from Kremers *et al*, 2005 [1] with permission). The intervention programme also involves awareness, attitude, self-efficacy, social influence, intention to change and energy balance related behaviours.

The effectiveness of the intervention programme is evaluated as shown in figure 2 using various consecutive outcomes as depicted in figure 3. In brief, data on body composition, blood pressure, diet and physical activity are collected at all four periods, whereas data on psychosocial determinants are gathered at baseline and after 6 and 12 months only. More details about the measurements are provided below.

#### Questionnaires

At baseline, data on demographical factors, such as education, date of retirement, and marital status are collected, as well as information on perceived health, smoking habits, use of hormone replacement therapy for women and drug use for high cholesterol levels, high blood pressure and diabetes mellitus.

Psychosocial determinants are assessed for all five identified behaviours (see figure 1) separately. Attitude, social support, norms and pressure, and self-efficacy expectations are determined based on commonly used constructs of the cognitive factors from the Theory of Planned Behaviour [51]. Intention to change and the stage of change for the five behaviours are also assessed; the assignment to stage of change being based on a combination of the Precaution Adoption Process Model [52,53] and the TransTheoretical Model [54]. Habit strength is assessed based on the Self-Report Habit Index [55] using three indicators of habit.

Data on dietary intake are collected using a self-administered semi-quantitative Food Frequency Questionnaire [56]. From these data total energy intake, total fat intake, saturated, mono- and poly-unsaturated fatty acids, cholesterol, total protein, total carbohydrate and alcohol consumption are derived. Fruit and vegetable intake (gram/day) is used to approximate fibre consumption. Portion size for energy dense foods is calculated as number of servings divided by the frequency of consumption.

Physical activity is assessed using the self-administered Dutch version of the Physical Activity Scale for the Elderly (PASE) [57]. The PASE assesses physical activity in the previous week in older people (aged 65–100 years) and specifically includes activities of the daily living, such as household activities [57,58]. The questionnaire assesses frequency and duration of activities at several intensities resulting in the PASE score that ranges from 0–400 with higher scores indicating greater activity levels [58].

The process evaluation is based on Rogers' diffusion of innovations-model [59] and data are collected at all follow-up measurements by using questionnaires monitoring the intervention delivery, participation, comprehension, satisfaction, level of use, and fidelity.

#### Anthropometry

Baseline physical examinations are conducted at the location of the pre-retirement workshops. Immediately after the one-year intervention participants are re-examined. To test the sustainability of the effects participants are measured again one year after the cessation of the intervention programme (see top part figure 2). Follow-up examinations are mostly performed at community health centres throughout the Netherlands, at the same time of day and by the same researcher compared to baseline.

Physical examinations are conducted between 11.00 am and 2.00 pm with participants wearing underwear only. Body weight is measured to the nearest 0.2 kg with an electric weigh-beam (SECA 840 scale & SECA 888; Vogel & Halke GmbH & Co KG, Hamburg, Germany) and body height to the nearest 0.1 cm with a mobile stadiometer (SECA 225; Vogel & Halke GmbH & Co KG, Hamburg, Germany). Waist circumference is measured at the midpoint between the lower rib and the iliac crest, hip circumference at the trochanter level, and thigh circumference immediately below the gluteal fold, upper arm circumference at the midst between the acromion and olecranon and calf circumference between the knee and ankle malleoli, with the leg at a 90° angle. Circumferences are measured twice to the nearest 0.1 cm with a plastic measuring tape. These anthropometric measurements are taken with participants in an upright position. Abdominal sagittal diameter is measured twice at the midst between the lower rib and iliac crest with participants in a supine position.

#### Body composition

Total body water is assessed by bioelectrical impedance measurements at 100 kHz by a tetra-polar, single-frequency device (BCM Controller, Data Input, Frankfurt, Germany) from which percentage total body fat is derived. In a subgroup randomly assigned from the clusters (at least n = 80, based on an additional sample size calculation using Fisher's transformation to determine ρ > 0.5) extended examinations are performed. Skinfold thickness are determined at the sites of biceps, triceps, sub scapula and supra iliaca. Total body scans are made using Dual Energy X-ray Absorptiometry (Lunar Radiation Corporation, Madison, WI, USA) from which relative amounts of fat in the abdominal region are derived.

#### Blood pressure

Blood pressure is assessed twice as an indicator of general health status, with participants in a supine position, using an automatic device (Omron).

### Statistical analysis

Analyses will be based on the intention-to-treat principle. Because of the cluster randomisation, we will use multilevel analyses (SAS PROC MIXED), with cluster as the random intercept. Baseline values of the dependent variable will be included as covariates. Furthermore, we plan to perform secondary analyses to explore intervention effects in subgroups of gender, education, body fatness and activity at latest job [60-62]. Finally, adherence to the intervention, defined as the self-reported use of the intervention materials (range 0–5), may be related to the outcome measures.

## Discussion

This study protocol presents the development of a low-intensity, multifaceted individually tailored energy balance programme and presents the design of the cluster randomised controlled trial to test the effectiveness of the programme. The intervention programme aims to prevent weight gain and in particular accumulation of abdominal fat mass in recent retirees. The content of the programme focuses on increasing awareness of energy balance and subsequently adapting behaviours according to participants' preferences.

To our knowledge, this intervention programme is unique because it applies energy balance strategies to a population of recently retired people. This population is particularly at risk for changes in daily routine physical activity and diet, because they leave the work force. If these changes are unfavourable they may lead to excessive body weight and accumulation of body fat. This is detrimental in a population that already has an elevated absolute risk of chronic diseases, such as diabetes mellitus and cardiovascular diseases [63]. Therefore, this programme may eventually reduce the risk for chronic diseases, resulting in a reduction of unhealthy life years and health care costs [64].

With respect to the development of intervention programme, some considerations should be made. First, we did not always have access to data representing the determinants of diet and physical activity of our target population. Instead we used information that was either valid for the total adult population or for the general middle-aged population. Furthermore, the intervention programme focuses on prevention of accumulation of abdominal fat and preserving muscle mass and not on reducing body weight. Ageing itself may lead to loss of muscle mass and gain of fat mass [15,16] and we wanted to avoid that participants would lose to much body weight, which may have negative effects at older age [63]. Thus, the programme stresses that that those intending to lose large amounts of body weight should consult their physician, and that overweight participants should not lose more than 5–10% of initial body weight.

Positive aspects of the intervention programme are that it covers both diet and physical activity, offers multiple feedback options instead of a 'one-size fits all' approach and is of low-intensity, all of which may benefit compliance [1].

In our study we focus on a sequence of consecutive outcome measurements. The main outcome is abdominal fat mass, which is measured by waist circumference and in a subgroup by Dual-Energy X-ray Absorptiometry. To estimate changes in muscle mass, we assess calf and upper-arm circumference. Since the menopause affects changes in body composition with ageing among females we collect data on menopausal status by questionnaire to be able to account for this.

We use questionnaires to evaluate the effects of the programme on dietary and physical activity behaviour. The advantage of questionnaires is that they are easily applicable in larger scale studies. To evaluate changes in physical activity, we use the Physical Activity Scale for the Elderly (PASE) [58]. The PASE is developed for an older age group and assesses household activities, daily activities and leisure time physical activity. The questionnaire is validated in Dutch older persons and the validity is moderate. To evaluate energy intake and total fat intake, we use a food frequency questionnaire validated for energy intake and fat intake [56]. To estimate changes in fibre we will use fruit and vegetables consumption and to approximate changes in portions, we will use the number of servings per months.

To conclude, transition to retirement seems a proper occasion to intervene with an energy balance programme. Such a programme may contribute to slowing down the increasing trend of overweight among retirees. Results from the trial are expected in 2007 and if effective and sustainable, the programme will be implemented in order to reach all 100,000 retirees per year in the Netherlands.

## Competing interests

The author(s) declare that they have no competing interests.

## Authors' contributions

AW, AJS, FJK and EGS are the principal investigators of the study, developed the concept and the design of the study. AW and JS drafted the manuscript. LK, TLSV and SPJK contributed to the design and content of the intervention programme. All authors read and approved the final manuscript.

## Pre-publication history

The pre-publication history for this paper can be accessed here:



## Figures and Tables

**Table 1:** Personal determinants, methods and description of materials provided to the intervention group.

**Personal determinants**	**Methods**	**Strategies**	**Description**
Awareness of own EBRBs^1 ^and of interaction between EBRBs^1^	Self-monitoring/self-evaluation; Feedback; Prompt; Information delivery.	Tools for personal evaluation; Active learning strategy.	**Box **(20*20*2.5 cm), sent by post: Instruction leaflet including a diary for activities and diet; Pedometer (advice to take 10.000 steps per day)^2^; Waist tape^3 ^to asses waist circumference, with colour indication; Instrument to easily assess the BMI; Fruit & vegetable consumption self-test, scratch test format^3^; Fat consumption self-test, scratch test format^3^; Calorie guide to provide information on the balance between calories of several food products and exercise (minutes walking, swimming and bicycling)^4^.
Knowledge of (own) EBRBs^1^.	Information delivery; Feedback; Individualised messages.	Internet.	Study website: • General information and more comprehensive information (after login) on diet, physical activity and the trial. • Access for 6 months to the Weight Co@ch^®5^, an interactive web-based programme providing goal setting on diet and/or physical activity based on individually assessed outcomes [50].
Knowledge of own BMI and standards; Positive attitude and high self-efficacy expectation with regard to weight gain prevention; Habit formation with regard to weight gain prevention.	Confrontation with personal risk; Individualised messages; Decisional balance; Skill training; Goal-setting.	Computer tailoring.	CD-ROM I, sent by post, including manual: Stage-matched feedback on BMI and statements on EBRB (yes/no), e.g. "Do you consciously watch less television to use that time to be physically active?" Note that it is stressed not to lose more than 5–10% of initial body weight, unless a physician is consulted.
Knowledge of own EBRBs^1^; Positive attitude and high self-efficacy expectations towards EBRBs^1^; Habit formation with regard to EBRBs^1^.	Feedback; Individualised messages; Decisional balance; Skill training; Goal-setting.	Computer tailoring.	CD-ROM II, sent by post, including manual. All feedback is provided in relation to the guidelines for the behaviour. **Physical activity**: minutes per week, based on frequency and duration [65]. **Fat consumption**: fat score based on fat consumption at cold and hot meals and snacks [66]. **Fibre consumption **based on consumption and preference for whole grain foods, fruits and vegetables. **Portion sizes **of energy dense foods during cold and hot meals, snacks and beverages. Photographs from the EPIC study are used to indicate different portion sizes with permission [67, 68]

^1^EBRBs = energy balance-related behaviours; ^2^type WA101 (Oregon Scientific); ^3^Netherlands Nutrition Centre, The Hague, the Netherlands; ^4^Netherlands Heart Foundation; ^5 ^Weight Co@ch is obtained from TNO Quality of Life.
